# Physician and Patient Predictors of Evidence-Based Prescribing in Heart Failure: A Multilevel Study

**DOI:** 10.1371/journal.pone.0031082

**Published:** 2012-02-21

**Authors:** Frank Peters-Klimm, Gunter Laux, Stephen Campbell, Thomas Müller-Tasch, Nicole Lossnitzer, Jobst-Hendrik Schultz, Andrew Remppis, Jana Jünger, Christoph Nikendei

**Affiliations:** 1 Department of General Practice and Health Services Research, University Hospital Heidelberg, Baden-Wuerttemberg, Germany; 2 Department of Psychosomatic and General Internal Medicine, University Hospital Heidelberg, Baden-Wuerttemberg, Germany; 3 Department of Cardiology and Pneumology, University Hospital Heidelberg, Baden-Wuerttemberg, Germany; Yale University School of Medicine, United States of America

## Abstract

**Background:**

The management of patients with heart failure (HF) needs to account for changeable and complex individual clinical characteristics. The use of renin angiotensin system inhibitors (RAAS-I) to target doses is recommended by guidelines. But physicians seemingly do not sufficiently follow this recommendation, while little is known about the physician and patient predictors of adherence.

**Methods:**

To examine the coherence of primary care (PC) physicians' knowledge and self-perceived competencies regarding RAAS-I with their respective prescribing behavior being related to patient-associated barriers. Cross-sectional follow-up study after a randomized medical educational intervention trial with a seven month observation period. PC physicians (n = 37) and patients with systolic HF (n = 168) from practices in Baden-Wuerttemberg. Measurements were knowledge (blueprint-based multiple choice test), self-perceived competencies (questionnaire on global confidence in the therapy and on frequency of use of RAAS-I), and patient variables (age, gender, NYHA functional status, blood pressure, potassium level, renal function). Prescribing was collected from the trials' documentation. The target variable consisted of ≥50% of recommended RAAS-I dosage being investigated by two-level logistic regression models.

**Results:**

Patients (69% male, mean age 68.8 years) showed symptomatic and objectified left ventricular (NYHA II vs. III/IV: 51% vs. 49% and mean LVEF 33.3%) and renal (GFR<50%: 22%) impairment. Mean percentage of RAAS-I target dose was 47%, 59% of patients receiving ≥50%. Determinants of improved prescribing of RAAS-I were patient age (OR 0.95, CI 0.92–0.99, p = 0.01), physician's global self-confidence at follow-up (OR 1.09, CI 1.02–1.05, p = 0.01) and NYHA class (II vs. III/IV) (OR 0.63, CI 0.38–1.05, p = 0.08).

**Conclusions:**

A change in physician's confidence as a predictor of RAAS-I dose increase is a new finding that might reflect an intervention effect of improved physicians' intention and that might foster novel strategies to improve safe evidence-based prescribing. These should include targeting knowledge, attitudes and skills.

## Introduction

Heart failure (HF) remains a deadly and costly, however treatable disease [1]–[3]. The clinical management of HF is complex and includes a repeated evaluation of the clinical course of the syndrome and its' comorbidities. Moreover it encompasses patient education, non-/pharmacological treatment, devices and surgery. Thus a coordinated and transdisciplinary approach is mandatory. Evidence-based pharmacological treatment, such as the use of renin angiotensin aldostererone inhibitors (RAAS-I) and betablockers (BB) requires the physician's competence in prescribing appropriate medications (indications vs contraindications) and step-wise up-titration while monitoring typical side-effects (i.e. hypotension, change in creatinine-clearance or potassium levels) during the subsequent trajectory of the syndrome [4]–[8].

Despite the consensus on clinical practice guidelines (CPG) that recommend the use of RAAS-I in target doses [8]–[10], there seems to be imperfect transfer into practice, especially in primary care. Current literature suggests that many patients actually, do not receive RAAS-I, mostly due to clinical and/or professional uncertainty or unawareness [11]. If prescribed, doses were titrated to only 50% of the target doses recommended in the CPGs [11], [12]. Understanding this gap between a physician's knowledge and his actual acting might therefore be essential for the development of strategies aiming to improve the care of HF patients [13].

In general, reasons for non-adherence to guideline recommendations can either be attributed to the knowledge and attitudes of physicians or might be due to external factors like specific reimbursement procedures or patient preferences [14]. Self-reported physician-related barriers to evidence based prescribing of HF medication include lack of knowledge or confidence [15]–[18], but these do not explain variance in treatment alone [18]. Usually, physician characteristics, as part of explorative studies, have been shown to impact the quality of care the patients receive. For example, working individually more than 15 years as a primary care physician has been correlated with non-prescription of RAAS-I [19]. Moreover a comparison between specialties revealed that primary care physicians use less diagnostic procedures and less evidence-based pharmacotherapy which was found to be explained only in part by patient characteristics [20].

However, many patients with heart failure have comorbidities that would have prevented inclusion in RCTs that have shown benefits in mortality [21], which reflects the complexity physicians face (especially in primary care) in the treatment of elderly, multimorbid patients [22]. Patient characteristics that have been found to be associated with the prescription of RAAS-I are age [12], [19], [23], gender [23], NYHA functional class [24] and comorbidity, such as hypertension [19], [24] and renal failure [12]. Even organizational aspects of health care systems may impact on prescribing patterns [25], irrespective of variations in guideline recommendations across Europe [26].

In order to explore the effect of physician and patient factors on RAAS-I dosage in patients with systolic heart failure, we used data collected as part of the Train-the-Trainer (TTT) trial (ISRCTN08601529) that compared the effectiveness of two specific medical education interventions for primary care physicians [27], [28]. Clinical trial design and data quality (with a pre-specified population of patients with systolic heart failure) offered the opportunity to study target dosing in the context of medical education. Results of the TTT trial showed that a multidisciplinary, complex intervention is superior to a single lecture focusing on evidence-based prescribing [27] as far as guideline adherence is concerned.

We therefore aimed to examine the interrelation of physician's knowledge and self-perceived competencies regarding RAAS-I as well as known patient-related barriers in clinical management with the actual practice of prescribing RAAS-I to HF patients further elucidating the individual impact of these factors.

## Methods

The Train-the-Trainer trial was a cluster-randomized multifaceted CPG implementation trial aimed at improving quality of life (QoL) of patients with HF by a mixed educational intervention for primary care physicians [27], [28]. After recruitment of physicians and enrolment of eligible patients and baseline patient assessment, physicians were randomized to either the intervention comprising multiple educational sessions plus pharmacotherapy feedback (TTT) or a lecture (Standard). After seven months (follow-up) a second patient assessment took place. The trial conformed to the principles outlined in the Declaration of Helsinki and was registered (ISRCTN08601529).

### Study Design and Objectives

**Figure 1:**
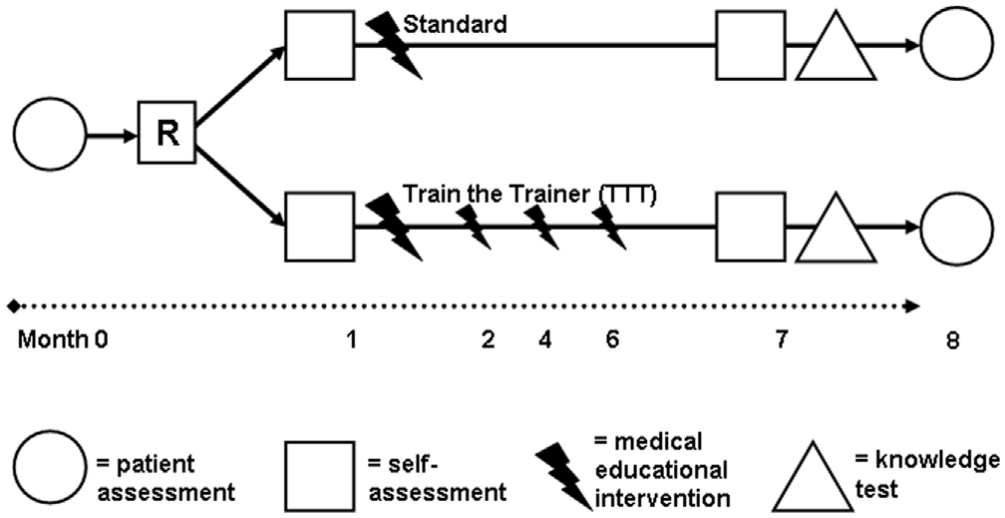
Trial design. (in chronological order:) Patient enrolment by primary care physicians and baseline clinical documentation (patient assessment), randomisation of physicians (with patients in clusters), physician self-assessment of competencies before (first) medical education intervention (either TTT or Standard), second physician self-assessment before unheralded knowledge test, follow-up patient assessment.

As part of the pre-specified evaluation of the secondary outcomes, self-perceived competencies (at baseline and at follow-up, i.e. one and seven months after randomization) and care-specific knowledge (at a seven month follow-up only) were assessed (see Figure 1). Our objective was to explore the simultaneous influence of pre-specified physician and patient factors on RAAS inhibitor dosage in a post-interventional cross-sectional design. Furthermore, the influence of distinct baseline variables was examined to verify the results (see Figure 1).

### Participants

Physicians were eligible for participation if they were certified as a primary care physician and practised as a SHI (statutory health insurance)-affiliated physician [28]. They recruited eligible patients using provided screening algorithms for case finding in electronic medical records. Eligibility criteria were adults (aged over 40 years) with left- or biventricular heart failure, NYHA functional class II–IV, with confirmed ejection fraction of 40% or less (e.g. by echocardiography), with stable symptoms at enrolment, and diagnosis of a chronic, irreversible HF at least 3 months prior to inclusion. We excluded patients with primary valvular heart diseases, hypertrophic obstructive/restrictive cardiomyopathy (HOCM/RCM), and people with a concomitant terminal illness, dementia or severe psychological illness. All participants gave written informed consent [28].

After an initiation visit, physicians collected and documented clinical data (general and cardiovascular history, actual clinical status, lab and other results, detailed medication, etc.) in case report forms (CRF) in their offices. They rated NYHA functional class according to the provided definition, and they documented aetiology, left ventricular ejection fraction (LVEF) or (if applicable) comorbidities from cardiologist and other specialist reports. Accordingly, to retrieve an estimate of patients' “morbidity burden” in addition to the documentation of single co-occurring medical conditions, physicians applied the Cumulative Illness Rating Scale (CIRS-G) [29] that measures the chronic medical illness (“morbidity”) burden while taking into consideration the severity of chronic diseases in 14 items representing individual body systems. The final score of the CIRS is the sum of each of the 14 scores, a higher score indicating higher impairment [range 0–56]. Actual laboratory results including creatinine and potassium were also recorded. We estimated the creatinine clearance using the formula by Cockroft and Gault [30]. Physicians documented prescribed drugs and daily doses in detail allowing further derivations. CRFs underwent a query management providing high data quality as part of the trial [28].

Physicians received their educational interventions at the medical university. Self-perceived competencies were assessed twice, before the (first) educational session and before an unheralded knowledge assessment that took place one month before follow-up (see Figure 1).

### Medical educational interventions

A framework guided us in the development of the educational interventions [31] which was based on a problem identification and general needs assessment [11], [17], [32] and a specific “needs assessment of targeted learners” (publication in preparation) and finally along the HF CPG [8].

The identified crucial topics of desired improvement, e.g. indication and management of evidence-based pharmacotherapy, detection and management of somatic psychological comorbid disorders, communication and organisational skills were formulated as specific learning goals (step 3). *Both* educational interventions were based on the same learning goals and included all relevant aspects of heart failure (epidemiology, definition, classification, diagnostic, therapeutic and management aspects of systolic and diastolic heart failure) and relevant psychosomatic aspects (comorbid depressive disorders and anxiety, health-related quality of life, and compliance). However, the educational methods, the intensity and the educators involved differed (details are reported elsewhere) [28]. This mix of methods, intensity and educators was part of the development and tailoring and due to the main intention of the project, i.e. improving health-related quality of life of patients with HF, an ambitious goal that was based on the assumption of “plenty” room of improvement in the care of patients [28]. Physicians from the Standard group received the CPG and a three-hour state-of-the-art lecture by a senior cardiologist with didactic expertise (AR). Physicians in the TTT group received a didactic (using different didactic formats), repeated (four sessions) and interdisciplinary (primary care physician, cardiologist and psychosomatic specialist: FPK, AR and TMT) educational intervention with a total duration of 16 hours (see Figure 1). Additionally, the TTT group received a pharmacotherapy feedback on the individual level of patients participating in the trial (from data of the baseline documentation) in the last session in month 6 [27].

### Procedures and Investigations

#### Outcome variable

Assessment of adherence to recommendations regarding RAAS-inhibitors was based on the current national clinical practice guideline that was used in the educational intervention [6]–[8] with minor national variations concerning substances (**, see below) and daily target doses due to differences in comparison to the European guidelines [4] [33]. The recommendations by product information were congruent with the guideline recommendations with the exception of Lisinopril (10 mg) and Losartan (100 mg in case of concomitant hypertension). In these cases, we decided to adhere to the guideline recommendation, because we wanted to assess the adherence to the guidelines, not to the product information. Substances were (daily target doses in brackets): ACE-inhibitors (ACE-I): Captopril (150 mg), Enalapril (20 mg), Lisinopril (20 mg), Ramipril (10 mg), Trandolapril (4 mg), Benazepril (20 mg)**, Fosinopril (35 mg)**, and Perindopril (4 mg)**. Angiotensin receptor blockers (ARB): Candesartan (32 mg), Valsartan (320 mg), and Losartan (100 mg)**.

#### Determinants

Our choice of variables to be analysed with respect to their predictive value was based on the literature and clinical expert experience [12], [15]–[19], [23], [24]: Considering the sample size we took pre-specified potential determinants, eight patient-related (i.e. patient age, gender, NYHA functional class at baseline, systolic and diastolic blood pressure, potassium level and creatinine clearance at follow-up, CIRS-G sum score [29] as measure for comorbidity and intervention group (Standard vs. TTT)), three physician-related variables (specific knowledge score [range 0–7], self-assessed frequency of prescription of RAAS-I [range 1–5] and global self-confidence in therapy of HF [range 0–100]).

The intervention team formulated multiple choice questions (MCQ) according to a blueprint accounting for the rules for fair and valid MCQ [34]. Depending on their contextual focus and origin, the formulation of the MCQ was assigned to the cardiologist and primary care researcher or to the psychosomatic specialist and primary care researcher. After *panel pre-review*, the 40 most appropriate questions from the 58 MCQ in the original version remained for the assessment: one correct answer represented one point. The ratio of cardiologic to psychosomatic MCQ remained 3 to 1. After the MCQ-test at the educational workshop, the MCQ were analysed in a *panel post-review* process for item difficulty, reliability and discriminative power. We pre-specified to allow only MCQ with a minimum discriminative power of r′>0.2 to remain in the test to shape the outcome measure to a valid, reliable and discriminative instrument (for details see Appendix S1). The application of objective criteria resulted in 26 remaining questions for final evaluation (20 with cardiologic and 6 with psychosomatic focus). The type of MCQ was “single best answer”. Cronbachs alpha of the overall MCQ-test with 26 questions (after the pre-specified item-reduction from 40 to 26) was 0.76 (, for the first 0.66). The subgroups of cardiologic and psychosomatic MCQ had a Cronbachs alpha of 0.72 and 0.58, respectively. To further focus on knowledge related to the use of RAAS inhibitors, we selected those 7 of the 20 cardiologic questions that were obviously related to this aspect, resulting in a specific knowledge score [0–7] (see also Appendix S1).

A self-developed questionnaire for self-perceived competency in the care of patients with HF was used. The questionnaire followed main learning objectives and included the self-rated *frequency* of used drugs and the related *self-confidence* using a Likert scale from 1–5, with low scores indicating a higher frequency or self-confidence. Participants were also asked to rate their *overall confidence* within the *therapeutic* domain of HF care using a visual analogue scale (VAS) [0–100], higher sores indicating a higher confidence. Participants completed this questionnaire before the (first) educational interventions at the medical university and before the knowledge assessment that took place at month seven (one month before patient follow-up, for illustration see Figure 1).

### Ethics

Ethical approval for the study was obtained from the institutional review boards of the medical faculty of the University of Heidelberg (252/2004) and of the medical association of the state of Baden-Wuerttemberg (M-049-05-f). Written informed consent was obtained from participating physicians and patients.

### Statistical methods (if applicable)

A logistic regression model was used for the outcome variable dosing of recommended RAAS inhibitors as the distribution of mean percentages of doses did not allow a linear model. Therefore, we treated the outcome variable as a dichotomous variable with (cut-off ≥50% of target dose). Dummy variables were built for ordinal variables (e.g. self-perceived confidence). We aggregated NYHA functional class I with II and III with IV accounting for the low number of observations in the classes I and IV. For the regression model, we imputed missing data using means of available data regarding self-rated frequency of prescription and overall self-confidence in therapy, in both there were missings in six cases. The model accounted for hierarchical clustering of the data, with patient variables and the outcome variable on level-1 and physician variables on level-two. The first model was validated by excluding variables with a p-value greater than 0.3. In other models, we replaced self-perceived competency variables measured at follow-up by those at baseline to verify further their predictive value. We used SPSS 15 for the analysis of descriptive data and SAS 9.2 (PROC GENMOD) for the two-level logistic regression analyses. As SAS outputs are reported in log odds ratios, we transformed these in odds ratios for better readability of the results.

## Results

### Physician and patient characteristics

**Figure 2:**
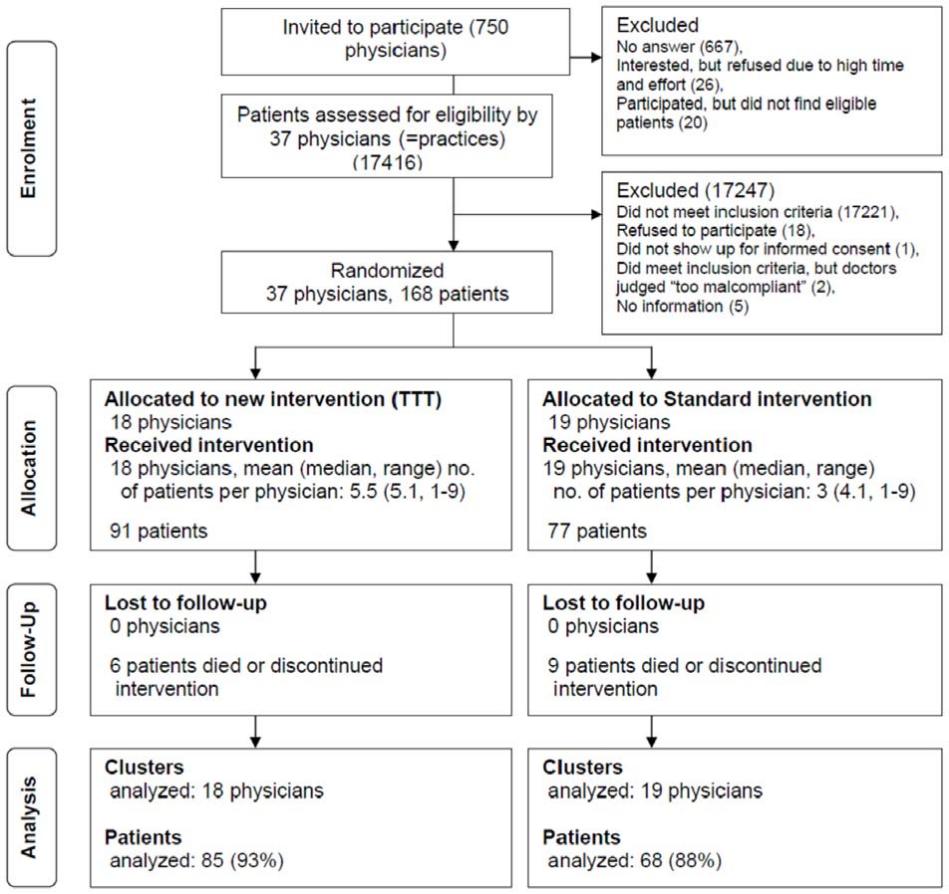
Flow of physicians and patients through the trial.

Figure 2 shows the flow of participating physicians and patients through the trial. Of the 750 physicians approached in a single mail-out, 37 ultimately participated and recruited 168 eligible patients between March and September 2005. Following patient recruitment, 18 physicians were randomised to the TTT group and 19 to the Standard group. At the patient level, 15 patients were lost to follow up (13 died and 2 were lost during the follow up). Therefore, for the outcome measure (dosage of recommended RAAS inhibitors) 153 (91%) patients were analysable.

Participating physicians were mainly male (81%), had a mean age of 50 and had on average been practicing as a primary care physician for 15 years (Table 1). They were practicing in individual practices in 46%, located in rural areas in 59%, and had a list size of less than 1500 per quarter in 59%.

**Table 1:** Baseline characteristics of all 37 participating primary care practices.

Physician and practice factors at baseline	TTT group (n = 18)	Control group (n = 19)
Female PCPs	3 (17)	4 (21)
Age of PCP in years (SD)	50 (9.4)	50 (5.9)
Certification of PCP since years (SD)	16 (11.4)	15 (7.2)
No. of PCPs (whole time equivalent)		
Single	9 (50)	11 (57.9)
Two	8 (44.4)	5 (26.3)
More than two	1 (5.6)	3 (25.8)
Location of practice		
Rural	13 (72.2)	9 (47.4)
Suburban	2 (11.1)	4 (21.1)
Urban	3 (16.7)	6 (31.6)
List size (patients per quarter)		
0–999	6 (33.3)	3 (15.8)
1000–1499	5 (27.8)	8 (42.1)
>1499	7 (38.9)	7 (36.8)
Participation in disease management programmes or quality circles	17/18 (94.4/100)	19/18 (100/94.7)

Values represent number (percentages) of physicians unless stated otherwise.

PCP: Primary Care Physician.

Of the enrolled 168 patients, 69% were male and their mean age was 68.8 years (Table 2). Table 2 shows patient characteristics, including NYHA functional class (II: 51% vs. III/IV: 49%) and a moderately impaired systolic function (LVEF 33±7%). In 44% of the cases coronary heart disease was the main cause for HF. Different medical conditions were prevalent, e.g. atrial fibrillation (19.6%), peripheral arterial disease (17.2%), COPD (19.6%) and Depression (23.2%). Others were diabetes (36.3%), hypertension (76.2%), and dyslipidemia (21.5%). Renal function was impaired in 23.8% of cases (GFR<50 ml/min). Mean (SD) potassium levels were 4.3 mmol/l, with 4 (2.4%) patients with hyperkalemia. Mean systolic and diastolic blood pressures were 131 and 77 mmHg. Physician-rated mean (SD) multimorbidity as indicated by CIRS-score was 23.4 (5.6). Most patients were treated with ACE inhibitors or angiotensin receptor blockers (90%), β-blockers (79%), aldosterone antagonists (29%), and many other drugs (see Table 2).

**Table 2:** Patient characteristics at baseline for groups (n = 168).

	TTT(n = 91)	Standard(n = 77)
Male sex	63 (69.2)	53 (68.8)
Mean (SD) age (years)	68.4 (10.6)	69 (9.5)
Living alone	27 (29.7)	22 (28.6)
NYHA-functional class (according to GP)		
II	44 (48.4)	41 (53.3)
III	46 (50.6)	33 (42.9)
IV	1 (1)	3 (3.9)
Mean (SD) LVEF	32.5 (7.1)(n = 79)	34.4 (6.5)(n = 64)
Ischemic Etiology	43 (47.3)	31 (40.3)
Mean (SD) duration (years) of CHF	5.6 (4.9)	5.8 (5.6)
Medical conditions		
Atrial fibrillation	21 (23.1)	12 (15.6)
PAD	15 (16.5)	14 (18.2)
Cerebrovascular disease	18 (19.8)	14 (18.2)
COPD	18 (19.8)	15 (19.5)
Depression	22 (24.2)	17 (22.1)
Cardiovascular risk factors		
Diabetes mellitus	32 (35.2)	29 (37.7)
Hypertension	68 (74.7)	60 (77.9)
Dyslipidemia	68 (74.7)	60 (77.9)
Creatinine-Clearance: Mean (SD) GFR (ml/min)*	74.1 (31.7)	66.5 (27.4)
GFR<50 ml/min	19 (20.9)	18 (23.4)
GFR<20 ml/min	2 (2.2)	1 (1.3)
Mean (SD) Kalium (mmol/l)	4.3 (0.6)	4.4 (0.5)
Hyperkalemia (K>5.5 mmol/l)	3 (3.3)	1 (1.4)
Mean (SD) Systolic Blood Pressure	130.9 (20.6)	130.2 (19.3)
Mean (SD) Diastolic Blood Pressure	76.7 (11.3)	76.1 (8.6)
Mean (SD) Comorbidity (CIRS-G)**	24.2 (6.0)	22.5 (4.8)
Drugs at baseline included:		
ACE inhibitor	69 (75.8)	61 (79.2)
ARB	15 (16.5)	10 (13.0)
ACE inhibitor or ARB	83 (91.2)	68 (88.3)
β-blocker	71 (78.0)	62 (80.5)
ACE inhibitor or ARB and β-blocker	65 (71.4)	57 (74.3)
Spirononolactone/Eplerenone	29 (31.9)	19 (24.7)
Loop diuretic	55 (60.4)	47 (61.0)
Thiazide diuretic	38 (41.8)	26 (33.8)
Cardiac glycoside	32 (35.2)	32 (41.6)
Nitrates (any)	17 (18.7)	12 (15.6)
Calcium channel blocker	9 (9.9)	7 (9.1)
Antiarrhythmic agents	8 (8.8)	5 (6.59
Aspirin	32 (35.2)	37 (48.1)
Statin	47 (59.7)	47 (51.6)
Oral anticoaculant	51 (56.0)	31 (40.3)
Insulin (any)	8 (8.8)	14 (18.2)
Oral antidiabetic	22 (24.2)	15 (19.5)

Values are numbers (percentages) of all patients unless stated otherwise.

NYHA, New York Heart Association; LVEF, Left ventricular ejection fraction; CHF, Chronic (systolic) heart failure; CHD, Coronary heart disease; PAD, Peripheral arterial disease; COPD, Chronic obstructive pulmonary disease.

*Estimation of the GFR according to the formula by Cockroft and Gault; ACE = angiotensin converting enzyme; ARB = angiotensin receptor blocker.

**CIRS-G, Cumulative illness (physician) rating scale, range 0–56, lower scores imply less impairment of 14 body systems.

Patient variables in bold were selected for the verification of their role as determinants of prescribing.

### Target variable and physician determinants

The mean (SD) percentage of daily target dose at follow-up for recommended RAAS-I was 47.0% (33.0), resulting in 90 patients (58.8%) that received equal or above 50% of recommended daily target doses.

Physicians' scores were in mean (SD; range) regarding specific knowledge 4.3 (1.4; 1–7) (n = 37), global self-confidence in the therapy at follow-up 79.2 (8.2; 66–92) (n = 37), and in frequency of and confidence in use of RAAS-I 1.4 (0.4; 1–2) and 1.6 (0.6; 1–3) (both n = 31).

For further results regarding between group comparisons see Appendix S1, table 4. Furthermore, participants evaluated both interventions with high levels of satisfaction (see Appendix S1, table 5).

### Predictors of prescribing

Patient age was the only patient-related variable with significant impact on dosing of recommended RAAS-I at follow-up. Patients with a higher age were less likely to be prescribed RAAS-I at doses of equal or more than 50% of target doses (odds ratio 0.95; 95% CI 0.92–0.99; p-value: 0.01). NYHA functional class showed a trend, i.e. patients with a higher NYHA class were less likely prescribed higher doses (odds ratio 0.63; 95% CI 0.38–1.05; p-value: 0.08) (see Table 3).

**Table 3:** Predictors of prescribing of ACE inhibitor or angiotensin receptor blocker (RAAS inhibitor) conforming with guideline recommendations at follow-up (n = 153).

Predictors of the final model	RAAS inhibitor ≥50% of daily target doseOdds ratio (95% CI), p-value*
Intercept	0.25 (0.00–145.15), 0.67
Treatment group (TTT vs. Standard)	0.63 (0.25–1.60), 0.33
Age	0.95 (0.92–0.99), 0.01
Gender (female vs. male)	0.68 (0.32–1.45), 0.32
NYHA functional class**	0.63 (0.38–1.05), 0.08
Specific knowledge related to pharmacotherapy (MCQ test)**	0.81 (0.62–1.17), 0.11
Self-assessed frequency of prescription of ACE inhibitors**	1.52 (0.64–3.64), 0.34
Self-assessed global self-confidence in therapy of CHF (VAS)**	1.09 (1.02–1.05), 0.01

*according to a two-level logistic regression model using PROC GENMOD accounting for clustering of the data.

**at follow-up.

Physician-related variables showed a potential impact of specific knowledge (related to pharmacotherapy), i.e. physicians with higher knowledge scores were less likely to prescribe higher doses (odds ratio 0.81; 95% CI 0.62–1.17; p-value: 0.11), this result, however, was not statistically significant. In contrast to self-assessed frequency, the global self-confidence in therapy of HF had a significant impact, i.e. the higher the global self-confidence, the higher were observed prescribed doses (odds ratio 1.09; 95% CI 1.02–1.05; p-value: 0.01) (see Table 3).

Neither multimorbidity (as measured by CIRS summary score) nor systolic/diastolic blood pressure, or renal function, or hyperkalemia were associated with prescription of higher doses of RAAS inhibitors (data not shown). So were neither global self-confidence in therapy, nor frequency of or confidence in the use of RAAS-I at baseline (data not shown).

## Discussion

### Summary of main findings

Optimizing evidence-based prescription in HF care is widely accepted as an appropriate and common performance measure. This study focused on primary care physicians' competency levels (before and after attending a CME event) and patient-related barriers as predictors of actual guideline-conform prescription of ACE inhibitors or angiotensin receptor blockers (RAAS inhibitors) at daily doses equal or more than 50% of target. Physicians' global self-confidence in therapy of HF positively determined while patient age negatively determined RAAS-I prescription towards target dose. Outcome-specific factual knowledge and a higher NYHA functional class showed a trend for a negative impact.

### Internal and external validity of the study

The fact that the parental trial was performed as a clinical trial promoted the internal validity [28]. For example, the defined patient population with confirmed symptomatic systolic heart failure is an essential aspect to assess evidence-based pharmacotherapy such as target dosing of RAAS-I. Similarly, patient variables derived from the trial documentation are characterized by a high validity. However, although developed with considerable time and effort, assessment of physician-related competence lacks validation and the study is relatively small in size which probably questions its power to detect significant predictors and to detect between group differences (see Appendix S1, table 4 competence outcomes).

A physician participation rate of 5% (see Figure 2) and some physician and patient characteristics may indicate that participants are not representative of the primary care physicians' population: “High time and effort” due to the trial's documentation and lack of eligible patients (both measures for a high internal validity) were the main reasons for the non-participation of physicians. Physicians' offices were larger than average (approx. 800/practice) and had a larger adoption rate of primary care-based disease management programs and peer review groups (approx. 53 and 30%). Baseline prescription rates of RAAS-I and betablockers revealed a superior guideline adherence as compared to other primary-care based studies, while being similar in comparison to studies conducted in secondary care [35], [36]. In our view, the observation of high prescription rates is rather based on the inclusion criterion of *objectified* systolic heart failure than a selection bias as it is the crucial indication to start RAAS-I. As primary-care-based studies in HF typically lack a predefined population (of systolic HF patients) prescribing rates are therefore lower. In fact, results of a primary care-based dutch study (10.6% confirmed systolic heart failure) suggest that an objectified systolic HF diagnosis, specific care in an HF clinic or referrals to a cardiologist are all associated with the prescription of ACE inhibitors [23].

### General interpretation in the context or current evidence

The understanding of how physician- and patient-related characteristics are associated with target dosing may foster future (educational) efforts to improve care. Some results are in line with previous findings such as age [12], [19], [23] and female gender being a negative patient-level predictor. We cannot explain the (non-significant) finding that a high NYHA score negatively determined RAAS dosing. A substudy of the IMPROVEMENT survey [11] showed that with higher NYHA scores the rates of ACE inhibitors increased [24]. This study, however, is not easy to compare as rates of ACE inhibitor prescription were high in our study and studies chose different outcomes (prescription versus target dosing rates). The patient population of the IMPROVEMENT survey shows another spectrum of patients, it was less defined regarding the left ventricular systolic dysfunction (LVEF<40% in 33% of patients in Germany and 27% in all countries) with another morbidity pattern of asymptomatic (12%), mild (34%), moderate (31%) and severe (10%) symptoms (percentages are given for the overall population). Therefore, in that study, NYHA stage in that population might indirectly reflect the type and advanced stage of heart failure, which, once assessed and confirmed crucially guide physicians' treatment decisions.

More interestingly, global self-confidence in the therapy of HF after (but not so prior to!) a CME event predicted target dosing, while conversely knowledge seemed to do the opposite. The first aspect is in line with literature that suggests that training may reduce variation between self- and external assessments [37]–[39], but the second is difficult to interpret: Qualitative studies suggest that increased confidence is associated with greater self-reported prescribing rates of ACE inhibitors [16]. It may be speculated that knowing more specific facts by itself increases the awareness of iatrogenic effects and might therefore induce reluctance in some physicians to increase the dose in elderly patients (primum nihil nocere!). This is reflected by various authors showing that primary care physicians did not use ACE inhibitors because of fear of side effects [15], [16], [40]. Psychological and organisational theory is being used to explain and to influence professional behaviour and might be helpful in relation to our findings, although there are virtually innumerable theories and constructs, without any final theory or model whatsoever [41]–[49]. Nevertheless, physicians' (post-interventional) self-confidence in our study might reflect the competence, motivation, and intention finally to prescribe higher dosages. Of note, it might also depend on personality traits as some physicians may have hesitated to use their newly acquired knowledge in case of elderly patients. However, our study did not identify objective predictive patient-related factors that reflect intolerance of up-titration. Therefore, final causal relations of this observation remain unclear.

Sinha et al. investigated physician characteristics in the context prescription of beta-blockers among patients with systolic HF, another important evidence-based treatment [50]: In their cross-sectional survey with a supplementary retrospective chart review they examined the association between primary care physician characteristics and both self-reported and actual prescription, results showed that physicians with teaching responsibilities and physicians with confidence in managing HF patients reported significantly higher rates of beta-blocker prescribing. However, only self-reported rates of prescribing were significantly associated with actual prescribing of beta-blockers among HF patients, not other physician characteristics, and the authors discussed a lack of power for further discrimination of the explanatory variables. Nevertheless, they concluded that self-confidence seems to play a role in beta-blocker prescribing in HF and should be targeted by teaching. Insofar the results of our study are in line with Sinha et al. and might be even stronger as they show the association of self-confidence and actual prescription.

### Limitations

Usually, determinants of evidence-based prescribing rely on explorative, secondary studies, which was the case in our study. However, our study has several limitations: It is relatively small in size, which prohibits further explanatory variables, whether physician- or patient-related [12], [19], [23], [24], or related to the organization of care [23]. As we were especially interested in the predictive role of different levels of physicians' competencies, we focused on these pre-specified variables while taking account for variables that play a direct role in medical decision making in this context. Further limitations are the use of self-developed and not completely validated assessments of physicians' competence. The use of imputation methodology in six observations might have affected the internal validity. Finally, the focus of this paper was not a primary outcome of the parental trial.

### Conclusions

Our findings replicated known barriers (such as patient age) to the prescription of RAAS-inhibitors according to actual guideline recommendations. Physicians' self-confidence and factual knowledge improved after the educational interventions, but only self-confidence became predictive of prescribing behaviour which might reflect an intervention effect of improved physicians' intention. Therefore, educational approaches that target knowledge, skills and attitudes (e.g. intentions) are required to further close the perceived performance gaps in complex behaviours such as safe evidence-based prescribing in elderly patients with heart failure. Further theory-driven research with comprehensive assessments using validated instruments and a larger dataset and sample is also recommended to further clarify the mechanisms of knowledge transfer into daily practice.

## Supporting Information

Appendix S1 **Knowledge, confidence and satisfaction, and MCQ-test.**
(DOC)
