# Supplementary material for: Physician and Patient Predictors of Evidence-Based Prescribing in Heart Failure: A Multilevel Study
Source: PLoS One. 2012 Feb 21;7(2):e31082. doi: 10.1371/journal.pone.0031082 (PMC3283612; doi:10.1371/journal.pone.0031082)
Supplement: Appendix S1 — Knowledge, confidence and satisfaction, and MCQ-test. (DOC) [file pone.0031082.s001.doc]

Appendix S1

### Knowledge, confidence and satisfaction*, and MCQ-test*

**Table 4**: Physician-related knowledge and self-assessed outcomes. Values are numbers (percentages) of all physicians (n=37) unless stated otherwise

| **Outcome** | **Time** | **TTT group**  (n=18) | **Standard group**  (n=19) | ANOVA (p-value)*** |
| --- | --- | --- | --- | --- |
| **Mean (SD) knowledge [range]** |  |  |  |  |
| Overall test result [0-26] | at follow-up | 17.2 (4.6)^****^ | 14.9 (3.7)^******^ | n.s. |
| Cardiologic domain [0-20] | at follow-up | 11.9 (4.1)^****^ | 10.8 (3.2)^******^ | n.s. |
| Psychosomatic domain [0-6] | at follow-up | 5.2 (1.0)^****^ | 4.1 (1.2)^******^ | **.046** |
| Questions related to ACE-inhibitors [0-7] | at follow-up | 4.4 (1.2)^****^ | 4.1 (1.7) ^******^ | n.s. |
| **Self-assessment** |  |  |  |  |
| Mean (SD) prescription of ACE inhibitors [range] |  |  |  |  |
| Frequency [1-5]* | at baseline | 1.6 (0.6)^*****^ | 1.7 (0.7)^******^ |  |
|  | at follow-up | 1.3 (0.5)^*****^ | 1.4 (0.5)^******^ | n.s. |
| Self-confidence [1-5]* | at baseline | 1.7 (0.7)^*****^ | 1.7 (0.8)^******^ |  |
|  | at follow-up | 1.5 (0.5)^*****^ | 1.7 (0.8)^******^ | n.s. |
| Mean (SD) global self-confidence in therapy of CHF [VAS**: range 0-100] | at baseline | 74.5 (8.7) | 73.8 (6.2)^*****^ |  |
|  | at follow-up | 84.6 (5.9) | 73.5 (6.1)^*****^ | **< .01** |

* low scores indicate a higher frequency or confidence

** VAS: Visual analogue scale, with higher scores indicate a higher self-confidence

*** To test for significant mean differences between the intervention and control groups in the MCQ-test and continuous variables of the characteristics of the GPs, one-way analyses of variance (ANOVA) with a subsequent post-hoc test (Bonferroni) were performed.

****n=17

***** n=16

****** n=15

**Table 5**: Satisfaction of all physicians (n=37) with the educational sessions of the „Train-the-trainer“(TTT)- and the state-of-the-art- (Standard) medical educational intervention unless stated otherwise

| Domain | Mean score (range) | | | | |
| --- | --- | --- | --- | --- | --- |
|  | **TTT group**  **(n=18)** | | | | **Standard group**  **(n=19)** |
|  | First session  (n=18) | Second session  (n=17)* | Third session  (n=13)** | Fourth  session  (n=14)*** | (n=14)**** |
| Information content | 1.2  (1-2) | 2.1  (1-3) | 1.5  (1-2) | 1.7  (1-5) | 1.4  (1-2) |
| Practical relevance | 1.4  (1-3) | 1.8  (1-3) | 1.5  (1-3) | 1.9  (1-4) | 1.9  (1-3) |
| Working atmosphere | 1.1  (1-2) | 1.1  (1-2) | 1.2  (1-2) | 1.4  (1-5) | 1.7  (1-4) |
| Didactic format | 1.4  (1-3) | 1.6  (1-3) | 1.3  (1-2) | 1.7  (1-5) | 1.6  (1-4) |
| Own participation | 2.0  (1-3) | 2.2  (1-3) | 1.9  (1-3) | 2.6  (1-4) | 2.1  (1-4) |
| Competence of educationalists | 1.1  (1-2) | 1.4  (1-2) | 1.2  (1-2) | 1.4  (1-5) | 1.0 |
| Organisation of the session | n.d. | 1.2  (1-2) | 1.1  (1-2) | 1.4  (1-5) | n.d. |
| Small group work | 1.3  (1-2) | n.a. | n.d. | n.a. | n.a. |
| Communication training | 1.3  (1-2) | n.a. | n.a. | n.a. | n.a. |
| Medication feedback  - [comprehensibility](http://dict.leo.org/ende?lp=ende&p=eL4jU.&search=comprehensibility) | n.a. | n.a. | n.a. | 1.4  (1-3) | n.a. |
| Medication feedback  - usefullness | n.a. | n.a. | n.a. | 1.9  (1-3) | n.a. |

Scores range from 1-5, with 1 indicating highest level of satisfaction; n.d.:not done / not asked; n.a.: not applicable

* one GP did not attend; ** two GPs did not attend, three missings (=no return of questionnaire, because GPs left earlier); *** one GP did not attend, three missings; **** two GPs attended at an alternative (similar) standard lecture without evaluation, three missings

**Multiple choice test**

Correct answers are in grey background.

To shape the outcome measure to a valid, reliable and discriminative instrument, very easy (P ≥0.85) and difficult questions (P ≤ 0.40) as well as questions with low discriminatory power (r’ ≤ 0.20) were cancelled.

The application of these criteria resulted in 26 remaining questions for final evaluation (20 with cardiologic, 6 with psychosomatic focus).

Within the cardiologic domain, specific questions related to indication and use of RAAS inhibitors are marked (questions 13, 15, 17, 18, 19, 23, 24), allowing one difficult and one easy question according to the abovementioned definition.

| **1.) Which statement regarding epidemiology of chronic heart failure is correct? (multiple answers are possible)** | | |
| --- | --- | --- |
| A) | Approximately 0.1 percent of the population in industrialised countries suffer from chronic heart failure. | **❑** |
| B) | A number of yearly newly diseased is 0.01 percent. | **❑** |
| C) | The life time risk for heart failure is approximately 20 % for both sexes (older than 40 years) . | **❑** |
| D) | Incidence and prevalence of heart failure are dependent on age. | **❑** |
| E) | Apporximately 0.1 percent of the population in industrialized countries suffer from chronic heart failure. | **❑** |

Item difficulty (p): p=0.433; Item discrimination (r`): r`=0.099

| 2.) Hypertension and coronary heart disease account for almost 75 percent of the population-related risk for heart failure. Which statement regarding the definition/etiology of heart failure is correct? | | |
| --- | --- | --- |
| A) | A pre-existing hypertension reduces the risk for heart failure status post myocardial infarction. | **❑** |
| B) | Heart failure is a syndrome with the final common pathway of multiple cardiac diseases presenting with specific organ symptoms. | **❑** |
| C) | Systematic studies with echocardiography in the Framingham study show that more than 90 percent of patients with heart failure have symptoms of heart failure. | **❑** |
| D) | Heart failure is defined as the failure of the heart to sufficiently supply the periphery of the body with blood to ascertain the metabolism of the tissue, in rest and under stress. | **❑** |
| E) | Chronic heart failure is characterised by a closed loop of hemodynamic compensatory mechanisms which counteract a further progression of the disease. | **❑** |

Item difficulty (p): p=0.489; Item discrimination (r`): r`=0.210

| **3.) A patient with chronic heart failure suffers from exertional dyspnea climbing the stairs to his flat 0n the 2rd floor. Following the usual classification the patients is in …** | | |
| --- | --- | --- |
| A) | NYHA functional class II | **❑** |
| B) | NYHA functional class III | **❑** |
| C) | in AHA stage B | **❑** |
| D) | …for classification a stress-test is obligatory | **❑** |
| E) | NYHA functional class IV | **❑** |

Item difficulty (p): p=0.689; Item discrimination (r`): r`=-0.009

| **4.) Which statement regarding the prognostic classification of heart failure is correct:** | | |
| --- | --- | --- |
| A) | NYHA functional stage 1 includes also symptomatic patients. | **❑** |
| B) | A newer classification of ACC/AHA 2001excludes risk constellations for the development of heart failure purposely. | **❑** |
| C) | The number of deaths due to heart failure is decreasing continuously since 1970. | **❑** |
| D) | Patients in NYHA functional class II/III under therapy with ACE inhibitors show a 1-year-mortality of approximately 10 percent in comparison to 50 percent in patients with terminal heart failure without such a therapy. | **❑** |
| E) | Patients with diastolic dysfunction have a bad prognosis similar to those with systolic heart failure. | **❑** |

Item difficulty (p): p=0.578; Item discrimination (r`): r`=0.115

| **5.) Which statement regarding the pathophysiology of chronic heart failure is not correct?** | | |
| --- | --- | --- |
| A) | The impairment of the cardiac cycle is primarily caused by myocardial damage. | **❑** |
| B) | The progression of heart failure is usually accelerated by the activation of neuro-endocrine processes. | **❑** |
| C) | Only hemodynamic factors are pathophysiologically relevant for ventricular remodeling. | **❑** |
| D) | Ventricular remodeling leads to increasing myocardial damage. | **❑** |
| E) | Neuroendocrine activation implies especially an activation of the renin-angiotensine-system. | **❑** |

Item difficulty (p): p=0.578; Item discrimination (r`): r`=0.115

| **6.) What are the most common two symptoms of heart failure in the elderly patients?** | | |
| --- | --- | --- |
| A) | Tachycardia and shortness of breath. | **❑** |
| B) | Shortness of breath and nocturia. | **❑** |
| C) | Reduced ability to exercise and shortness of breath. | **❑** |
| D) | Falls and fatigue. | **❑** |
| E) | Tiredness and atrial fibrillation. | **❑** |

Item difficulty (p): p=0.600; Item discrimination (r`): r`=0.282

| **7.) Which answer is incorrect? The determination of BNP for the diagnosis of heart failure is helpful specifically in elderly patients, because …** | | |
| --- | --- | --- |
| A) | patients frequently have a changed perception of symptoms and are frequently multimorbid. | **❑** |
| B) | the negative predictive value of the BNP-test is excellent and therefore, with a low BNP value, a relevant heart failure can be excluded. | **❑** |
| C) | a value of BNP more than 50 pmol/l is already pathologic. | **❑** |
| D) | also a diastolic dysfunction of the left ventricle leads to an albeit slightly increased level of BNP. | **❑** |
| E) | it helps in the differential diagnosis (of dyspnea) of COPD (without right ventricular load) and heart failure. | **❑** |

Item difficulty (p): p=0.400; Item discrimination (r`): r`=0.237

| **8.) Which diagnostic measure should be taken at minimum to validate the diagnosis of heart failure?** | | |
| --- | --- | --- |
| A) | Coronary angiography | **❑** |
| B) | Myocardial biopsy | **❑** |
| C) | Echocardiography | **❑** |
| D) | Determination of BNP | **❑** |
| E) | Treadmill test | **❑** |

Item difficulty (p): p=0.711; Item discrimination (r`): r`=0.115

| **9.) Which statement regarding heart failure is incorrect?** | | |
| --- | --- | --- |
| A) | Sinustachycardia and atrial fibrillation can be found in the ECG. | **❑** |
| B) | Repolarisation disorders or Q-waves can be found in the ECG. | **❑** |
| C) | A left bundle branch block can be found in the ECG. | **❑** |
| D) | A normal ECG renders the diagnosis of heart failure improbable. | **❑** |
| E) | Certain changes in the ECG directly lead to the diagnosis of heart failure. | **❑** |

Item difficulty (p): p=0.556; Item discrimination (r`): r`=0.045

| **10.) Regarding the diagnostic workup of systolic heart failure the following statement is correct:** | | |
| --- | --- | --- |
| A) | History and physical examination are rarely diagnostic | **❑** |
| B) | ECG and radiography of the thorax show pathognomonic findings in non-ischemic cardiomyopathy. | **❑** |
| C) | Echocardiography is of minor diagnostic value. | **❑** |
| D) | The markers BNP and NT-proBNP can be helpful to exclude left ventricular impairment in symptomatic patients. | **❑** |
| E) | If a chronic coronary heart disease is suspected a timely coronary angiography without prior ischemic diagnostic is obligatory. | **❑** |

Item difficulty (p): p=0.556; Item discrimination (r`): r`=0.224

| 11.) What is the definition of diastolic heart failure? Please name the three most important elements of the triad. | | |
| --- | --- | --- |
| A) | Peripheral edema, dyspnea, left ventricular hypertrophy. | **❑** |
| B) | Clinical signs of heart failure, impaired ejection fraction, left ventricular hypertrophy. | **❑** |
| C) | Nocturia, peripheral edema, neck vein distension. | **❑** |
| D) | Clinical signs of heart failure, normal systolic function, increase of diastolic filling pressure. | **❑** |
| E) | Increase of diastolic filling pressure, decreased A-wave, clinical signs of heart failure. | **❑** |

Item difficulty (p): p=0.489; Item discrimination (r`): r`=0.441

| **12.) Which of the following statements is incorrect? The BNP-Value is meaningful for the ……** | | |
| --- | --- | --- |
| A) | Prognosis of systolic heart failure. | **❑** |
| B) | Diagnosis of diastolic heart failure. | **❑** |
| C) | Differential diagnosis regarding cardiac or pulmonary causes of dyspnea. | **❑** |
| D) | Differentiation between systolic and diastolic heart failure. | **❑** |
| E) | Follow up of heart failure patients. | **❑** |

Item difficulty (p): p=0.511; Item discrimination (r`): r`=0.162

| **13.) Which statement regarding the long-term therapy of chronic heart failure (HF) is correct?** | | |
| --- | --- | --- |
| A) | You cannot alter the prognosis with a long-term therapy of HF. | **❑** |
| B) | Patients remaining symptomatic under a regimen of ACE inhibitors and beta-blockers, may benefit from an additional angiotensin receptor blocker. | **❑** |
| C) | You should not prescribe combinations of therapies. | **❑** |
| D) | Every patient with HF should receive digoxin or digitoxin. | **❑** |
| E) | To improve the prognosis diuretics are indicated in most stages of HF | **❑** |

Item difficulty (p): p=0.667; Item discrimination (r`): r`=0.240

| **14.) Which statement is incorrect? (multiple answers possible)**  **ACE inhibitors …** | | |
| --- | --- | --- |
| A) | lead to coughing as the most prominent side effect in 25-30 % of patients with heart failure (according to studies). | **❑** |
| B) | decrease cardiac remodeling induced by neuroendocrine activation. | **❑** |
| C) | reduce the mortality of chronic heart failure. | **❑** |
| D) | should be prescribed in heart failure only at low dosages. | **❑** |
| E) | should be started in low dosages and up-titrated slowly. | **❑** |

Item difficulty (p): p=0.478; Item discrimination (r`): r`=-0.059

| **15.) Which statement regarding treatment of chronic heart failure is correct?** | | |
| --- | --- | --- |
| A) | Betablockers are not indicated because of their negative inotrope effect. | **❑** |
| B) | Betablockers are only indicated in patients with coronary heart disease. | **❑** |
| C) | Betablockers are contraindicated in HF patients with comorbid chronic obstructive pulmonary disease (COPD). | **❑** |
| D) | The choice of a specific betablocker is of no concern because the „class effect” prevails. | **❑** |
| E) | Betablockers added to ACE inhibitors decrease mortality and hospital admission rates in patients in NYHA functional stage II-IV. | **❑** |

Item difficulty (p): p=0.889; Item discrimination (r`): r`=0.346

| **16.) Which of the following statements ist incorrect?**  **Aldosterone antagonists …** | | |
| --- | --- | --- |
| A) | can induce clinically relevant hypokalemias. | **❑** |
| B) | have antihypertrophic effects. | **❑** |
| C) | counteract the development of myocardial fibrosis. | **❑** |
| D) | significantly reduce mortality in the treatment of systolic heart failure. | **❑** |
| E) | need to be tested in the setting of diastolic heart failure. | **❑** |

Item difficulty (p): p=0.476; Item discrimination (r`): r`=0.255

| **17.) Which statement regarding ACE inhibitors in treatment of heart failure is correct?** | | |
| --- | --- | --- |
| A) | ACE inhibitors decrease hospital admission rates and improve symptoms in patients in NYHA functional class II-IV, but not mortality. | **❑** |
| B) | ACE inhibitors do not slow the progression of heart failure in asymptomatic patients with left ventricular systolic dysfunction. | **❑** |
| C) | Patients with symptomatic heart failure post infarction do not benefit from ACE inhibitors. | **❑** |
| D) | Starting ACE inhibitors in low doses lead to fewer side effects. | **❑** |
| E) | ACE inhibitors do not impact on the incidence of strokes. | **❑** |

Item difficulty (p): p=0.511; Item discrimination (r`): r`=0.201

| 18.) Which statement regarding betablockers in the treatment of heart failure is correct? | | |
| --- | --- | --- |
| A) | Betablockers can already be given when signs of cardiopulmonary congestion persist or during the weaning of intravenous inotropic agents. Würde ich so nicht unterschreiben, da wir es täglich auf Intensiv durchaus so machen | **❑** |
| B) | Betablockers with intrinsic sympathomimetic activity should especially be administered in heart failure. | **❑** |
| C) | Betablockers are contraindicated in chronic heart failure. | **❑** |
| D) | In the longterm one should aim for recommended target doses or target heart rate (50-60 bpm) as the improvement of the ventricular function seems to depend on an „effective level“. | **❑** |
| E) | In case of intolerability or contraindications for betablocker, combinations of an ACE inhibitor with an aldosterone receptor blocker are contraindicated. | **❑** |

Item difficulty (p): p=0.489; Item discrimination (r`): r`=0.245

| 19.) Which of the following statements regarding diuretics in heart failure is correct? | | |
| --- | --- | --- |
| A) | There are many randomised, prospective trials regarding the use of diuretics in congestive heart failure. | **❑** |
| B) | As monotherapy in stable heart failure, diuretics are recommended prior to ACE inhibitors. | **❑** |
| C) | The individual choice of a diuretic drug does not dependent on the renal function. | **❑** |
| D) | Intravenous Diuretics are more effective than their equivalent oral doses, especially in severe clinical conditions. | **❑** |
| E) | The aldosterone antagonist spironolactone is the preferred medication in patients in lower stages of heart failure. | **❑** |

Item difficulty (p): p=0.400; Item discrimination (r`): r`=0.473

| 20.) A 70 year-old patient in NYHA functional class III, presents with a known left ventricular dysfunction (LVEF = 40%) without signs of fluid retention, normal potassium and renal function. Treatment with an ACE-Inhibitor and a diuretic is tolerated reasonably well. To optimise pharmacotherapy you preferably add the following drug: | | |
| --- | --- | --- |
| A) | Digoxine. | **❑** |
| B) | An angiotensine receptor blocker. | **❑** |
| C) | An aldosterone antagonist. | **❑** |
| D) | Hydralazine und Isosorbiddinitrate. | **❑** |
| E) | A betablocker. | **❑** |

Item difficulty (p): p=0.889; Item discrimination (r`): r`=0.019

| 21.) The abovementioned 70 year-old patient shows in a control of the metabolic situation a Type 2 diabetes mellitus with a HBA1c of 8,1 % and a BMI of 31. Which statement is incorrect? | | |
| --- | --- | --- |
| A) | In renal insufficiency (Creatinine > 1,4 mg/dl), metformin is contraindicated because of a higher risk of lactate acidosis. | **❑** |
| B) | A slight to moderate systolic impairment is an absolute contraindication for the prescription of metformin. | **❑** |
| C) | Oral antidiabetics as monotherapy lower HBA1C for about 0,5-1 %. | **❑** |
| D) | The patient qualifies for a therapy with metformin, because of moderate heart failure, normal renal function and obesity. | **❑** |
| E) | Dietetic measures and adequate physical exercise are essential in this patient. | **❑** |

Item difficulty (p): p=0.822; Item discrimination (r`): r`=-0.091

| **22.) Your patient is 44 years old and has a dilative cardiomyopathy. Being on a medication with beta blocker, ACE inhibitor and spironolactone the patient is free of symptoms in daily routine, although he avoids physical stress. He is hospitalized on a surgical ward because of a nasal fracture after a fall at home. The consulted internist determines the BNP serum level that amounts to 1300 pg/ml. Seeing this patient one week after hospital discharge, he complains about sleep disturbances due to shortness of breath and about swollen legs. Which taken action is incorrect?** | | |
| --- | --- | --- |
| A) | I replace NSAR by acetaminophen | **❑** |
| B) | I start a diagnostic work-up for the cause of falling. | **❑** |
| C) | I determine BNP emergently. | **❑** |
| D) | I extend the actual therapy by a loop diuretic. | **❑** |
| E) | I instruct the patient about weight control, fluid restriction and self management if weight increases. | **❑** |

Item difficulty (p): p=0.733; Item discrimination (r`): r`=0.043

| **23.) (The abovementioned patient with dilatative cardiomyopathy) The patient is in NYHA functional class III, blood pressure 105/80 mmHG, actually no clinical signs of cardiac deterioration, normal creatinine, high-normal potassium. The ECG shows a left bundle-branch block. The holter ECG (24 h) does not show signs of rhythm abnormalities. The referral for echocardiography results in a globally impaired ventricular function (LVEF = 20 %)**  **Which statements/recommendations can be expected from the consulting cardiologist?**  **(multiple answers are possible)** | | |
| --- | --- | --- |
| A) | The patient received an additional angiotensine receptor blocker | **❑** |
| B) | The patient should be evaluated for the indication of a Cardiac Resynchronisation Therapy (CRT). | **❑** |
| C) | The patient should be evaluated for heart transplantation. | **❑** |
| D) | The patient should receive Phenprocoumon (oral vitamin k antagonist). | **❑** |
| E) | Regular controls of the serum potassium levels are essential under the abovementioned pharmacotherapy. | **❑** |

Item difficulty (p): p=0.144; Item discrimination (r`): r`=0.386

| **24.) A sixty-year old ischemic heart failure patient (LVEF = 20%) without angina pain presents in your office for a follow up. The maximal oxygen uptake VO2 max. is 12 ml/kg/min. Actual blood pressure (sitting) is 110/75 mm Hg, heart rate 90 bpm. Actual medication: Lisinopril (Zestril® 2,5mg/Tag), bisoprolol (Concor® mite 5 mg/Tag). What action is not correct?** | | |
| --- | --- | --- |
| A) | I try to check the patients’ medical record and history. I am especially interested whether the patient has undergone a coronary angiography and a test of myocardial viability of the heart. | **❑** |
| B) | I titrate the medication: Daily target doses of Zestril® and Concor® are 35 mg and 10 mg. I consider oral anticoagulation and an aldosterone antagonist. | **❑** |
| C) | Titrating Zestril® and Concor® I regularly assess the hydratation/volemia and avoid hypovolemic states due to diuretics. | **❑** |
| D) | I regularly check electrolytes, liver enzymes and the creatinine. | **❑** |
| E) | A repeated spiroergometry confirms a VO2-max. of 12 ml/kg/min meaning that the patient is definitively no candidate for heart transplantation . | **❑** |

Item difficulty (p): p=0.644; Item discrimination (r`): r`=0.378

| **25.) Which statement is incorrect? Potential precipitating factor of a diastolic heart failure is ...** | | |
| --- | --- | --- |
| A) | …a myocardial ischemia. | **❑** |
| B) | …uncontrolled hypertension. | **❑** |
| C) | …atrial fibrillation. | **❑** |
| D) | …a sinus bradycardia. | **❑** |
| E) | …an increased uptake of fluids. | **❑** |

Item difficulty (p): p=0.289; Item discrimination (r`): r`=0.109

| **26.) Which statement regarding diastolic heart failure is correct?** | | |
| --- | --- | --- |
| A) | Clinical guidelines regarding the therapy are based on robust evidence. | **❑** |
| B) | The prevalence increases with increasing age. | **❑** |
| C) | Men are affected more frequently than women. | **❑** |
| D) | The mortality is double than in systolic heart failure. | **❑** |
| E) | Most common cause is aortic stenosis. | **❑** |

Item difficulty (p): p=0.667; Item discrimination (r`): r`=0.546

| **27.) Which echocardiographically determined sign/stage of diastolic abnormality has the worst prognosis?** | | |
| --- | --- | --- |
| A) | Pseudo-normalisation. | **❑** |
| B) | Reversible restriction. | **❑** |
| C) | Decelerated relaxation. | **❑** |
| D) | The constriction. | **❑** |
| E) | Irreversible restriction. | **❑** |

Item difficulty (p): p=0.622; Item discrimination (r`): r`=0.029

| **28.) Which statement regarding left ventricular *diastolic* heart failure is incorrect?** | | |
| --- | --- | --- |
| A) | Systolic and diastolic heart failure are equally prevalent. | **❑** |
| B) | The ejection fraction determined by echocardiography is reduced. | **❑** |
| C) | The data base regarding the therapy of diastolic heart failure is not strong. | **❑** |
| D) | In hypertension normalisation of blood pressure is essential | **❑** |
| E) | Diagnostic evaluation and therapy should be done in collaboration on with a cardiologist. | **❑** |

Item difficulty (p): p=0.333; Item discrimination (r`): r`=0.487

| 29.) Regarding Cardiac Resynchronisation Therapy (CRT) which of the following statement is correct: | | |
| --- | --- | --- |
| A) | A right bundle branch block is a typical indication for CRT | **❑** |
| B) | Heart failure stages NYHA I and II are a typical indication for CRT | **❑** |
| C) | EF of 35 to 50 % is sufficient to indicate CRT therapy. | **❑** |
| D) | CRT in addition to optimal medication therapy can decrease mortality and hospitalisation rates in about 20 to 30 percent. | **❑** |
| E) | A combination of CRT with ICD does not reduce further mortality. | **❑** |

Item difficulty (p): p=0.844; Item discrimination (r`): r`=0.152

| 30.) Regarding ICD-Therapy in heart failure which of the following statements is correct: | | |
| --- | --- | --- |
| A) | Ventricular arrhythmias are of only of minor significance as a cause of death in heart failure patients. | **❑** |
| B) | ICD systems are similarly effective to medical therapy strategies in the prevention of sudden cardiac death. | **❑** |
| C) | Following MADIT II patients with non-ischemic cardiomyopathy benefit from ICD therapy. | **❑** |
| D) | ICD systems are only effective in secondary prevention. | **❑** |
| E) | ICD therapy yields an approximate 30% reduction of mortality in post-infarct patients presenting with an EF of 30% within a 2 years follow up. | **❑** |

Item difficulty (p): p=0.622; Item discrimination (r`): r`=-0.133

| 31.) What is the prevalence of depressive disorders in heart failure patients in ambulatory care needing treatment? | | |
| --- | --- | --- |
| A) | Approx. 2 %. | **❑** |
| B) | Approx .10 %. | **❑** |
| C) | Approx. 20 %. | **❑** |
| D) | Approx. 50 %. | **❑** |
| E) | There is no reliable information on this. | **❑** |

Item difficulty (p): p=0.467; Item discrimination (r`): r`=-0.079

| 32.) Cardinal symptoms of a depressive disorder are: | | |
| --- | --- | --- |
| A) | Depressed mood and markedly diminished interest. | **❑** |
| B) | Fatigue and loss of energy. | **❑** |
| C) | Diminished ability to think or concentrate and decreased appetite. | **❑** |
| D) | Excessive rumination and worries. | **❑** |
| E) | Insomnia during the night and fatigue during the day. | **❑** |

Item difficulty (p): p=0.467; Item discrimination (r`): r`=0.075

| 33.) The following statement regarding depressive disorders in heart failure patients is correct: | | |
| --- | --- | --- |
| A) | They have nearly no negative influence on hospitalisation rate and mortality. | **❑** |
| B) | In anxiety disorders they have a negative influence on hospitalisation rate and mortality. | **❑** |
| C) | They have a negative impact on hospitalisation rate, but not on mortality. | **❑** |
| D) | They have a markedly negative influence on hospitalisation rate and mortality. | **❑** |
| E) | They have a negative influence on mortality, but not on hospitalisation rate. | **❑** |

Item difficulty (p): p=0.422; Item discrimination (r`): r`=0.058

| **34.) In heart failure patients with depressive disorders the treating physician should …..** | | |
| --- | --- | --- |
| A) | … in case of concrete suspect of existing suicidal ideation only ask hereafter if a patient himself raises this topic. | **❑** |
| B) | … raise the topic of suicidal tendency only if patients have a history of attempted suicides. | **❑** |
| C) | … always assess suicidal ideation. | **❑** |
| D) | … not request suicidal ideation in order not to stimulate the intention of a suicide attempt. | **❑** |
| E) | … let psychiatrists request suicidal ideation exclusively. | **❑** |

Item difficulty (p): p=0.911; Item discrimination (r`): r`=0.118

| **35.) Which of the following statements regarding anti-depressive pharmacotherapy in patients with cardiac disease is correct:** | | |
| --- | --- | --- |
| A) | Especially in patients with psychomotor agitation or sleep disturbances tri- or tetra-cyclic anti-depressive medication is treatment of first choice. | **❑** |
| B) | Pharmacotherapy is more effective than psychotherapy and should therefore be preferred in the treatment of depressive disorders. | **❑** |
| C) | In patients with heart failure one should abstain from anti-depressive medication because of pre-existing polypharmacy. | **❑** |
| D) | SSRI (Selective Serotonin Re-uptake Inhibitors) have no major cardiac side effects according to the current state of knowledge. | **❑** |
| E) | In anti-depressive pharmacotherapy neuroleptic medication (for example “Depot-Fluspirilen”) should be considered because of there long-term anti-depressive effect. | **❑** |

Item difficulty (p): p=0.733; Item discrimination (r`): r`=0.277

| **36.) Which statement regarding anti-depressive pharmacotherapy with St. John´s wort in patients with cardiac disease is correct?** | | |
| --- | --- | --- |
| A) | As phytopharmacytical product St. John´s wort is not known for major interactions with other drugs. | **❑** |
| B) | By induction of liver enzymes effective drug levels can decrease, for example those of Phenprocoumon and Digoxine. | **❑** |
| C) | The interaction with immunosuppressive drugs is measurable under laboratory circumstances, but is not clinically relevant. | **❑** |
| D) | There is a clearly defined target dose with a well known efficacy. | **❑** |
| E) | Especially in cardiac diseases with multiple drugs (polypharmacy) St. John´s wort is the anti-depressive drug of first choice. | **❑** |

Item difficulty (p): p=0.822; Item discrimination (r`): r`=-0.091

| 37.) A generalized anxiety disorder is characterised by the following symptoms ….. | | |
| --- | --- | --- |
| A) | Nightmares, flash-backs and hypervigilance. | **❑** |
| B) | Nervousness, anxiousness and excessive worries. | **❑** |
| C) | Avoidance of specific situations and objects. | **❑** |
| D) | The compulsion to repeatedly take specific actions just to make sure that something has been organized appropriately. | **❑** |
| E) | The impulse to follow certain trains of thoughts to avoid frightening situations. | **❑** |

Item difficulty (p): p=0.489; Item discrimination (r`): r`=0.264

| **38.) In patients with heart failure and ICD (Implantable Cardioverter Defibrillator) anxiety and panic disorders are common. Which of the following statements regarding therapy is correct:** | | |
| --- | --- | --- |
| A) | Group psychotherapy is well evaluated and established in psychotherapy. | **❑** |
| B) | As the underlying heart failure is not reversible, long-term therapy with benzodiazepines is justified. | **❑** |
| C) | SSRI (Selective Serotonin re-uptake inhibitors) should not be given as anxiolytic therapy. | **❑** |
| D) | Psychodynamic therapies are the most effective form of psychotherapy in the treatment of panic disorder. | **❑** |
| E) | Elements of behavioural therapy can be used successfully for the maintenance or extension of area of action. | **❑** |

Item difficulty (p): p=0.689; Item discrimination (r`): r`=0.340

| **39.) The compliance with medication by patients with heart failure is determined by the following fact:** | | |
| --- | --- | --- |
| A) | The number of different drugs has no influence on drug adherence. | **❑** |
| B) | Even if patients with depressive disorders prefer a psycho-pharmacological therapy to psychotherapy, psycho-pharmacotherapy should be avoided to maintain the number of drugs as low as possible. | **❑** |
| C) | More than 5 different drugs are associated with a strong decrease of adherence. | **❑** |
| D) | Informations concerning medication given to the patients should be reduced to a minimum to avoid fear of side effects. | **❑** |
| E) | Tablet aids should not be used as they prevent a handling with the medication under once own authority. | **❑** |

Item difficulty (p): p=0.911; Item discrimination (r`): r`=0.311

| 40.) Following statement regarding health-related quality of life of heart failure patients is correct: | | |
| --- | --- | --- |
| A) | Quality of life depends on the NYHA functional class. | **❑** |
| B) | The quality of life clearly correlates with LVEF. | **❑** |
| C) | Elderly patients show a diminished quality of life. | **❑** |
| D) | Male patients show a lower quality of life than female patients. | **❑** |
| E) | The quality of life is independent of the prevalence of a depressive disorder. | **❑** |

Item difficulty (p): p=0.711; Item discrimination (r`): r`=0.330
